# God and the Doctor We Alike Adore

**Published:** 1897-02

**Authors:** Geo. Duffield

**Affiliations:** Detroit


### "GOD AND THE DOCTOR WE ALIKE ADORE."

DETROIT, January 9, 1897.

*Editors Atlanta Medical and Surgical Journal:*

The verse in the back of your JOURNAL for January, 1897, which you ask for the author of, is from Goldsmith. It is a familiar quotation.

Yours truly,

GEO. DUFFIELD.

---

CATGUT may be rendered aseptic and even antiseptic without complicated apparatus by the following simple method: Wind the gut on reels. Shake the full reels in a jar of ether to remove the fat. Soak them in ten per cent. carbolic made with alcohol for from six to twenty-four hours according to the thickness of the gut. Now place the reels in pure alcohol and the catgut is ready for use, and will be found to have lost little or none of its tensile strength.—*International Journal of Surgery.*

